# Location of the Cell Adhesion Molecule “Coxsackievirus and Adenovirus Receptor” in the Adult Mouse Brain

**DOI:** 10.3389/fnana.2020.00028

**Published:** 2020-06-04

**Authors:** Amani Wehbi, Eric J. Kremer, Iria G. Dopeso-Reyes

**Affiliations:** Institut de Génétique Moléculaire de Montpellier, CNRS, Université de Montpellier, Montpellier, France

**Keywords:** coxsackievirus and adenovirus receptor, adult neurogenesis, rostral migratory stream, hippocampus, extracellular matrix, synaptogenesis, cell adhesion molecules, canine adenovirus type 2 vector

## Abstract

The coxsackievirus and adenovirus receptor (CAR) is a single-pass transmembrane cell adhesion molecule (CAM). CAR is expressed in numerous mammalian tissues including the brain, heart, lung, and testes. In epithelial cells, CAR functions are typical of the quintessential roles of numerous CAMs. However, in the brain the multiple roles of CAR are poorly understood. To better understand the physiological role of CAR in the adult brain, characterizing its location is a primordial step to advance our knowledge of its functions. In addition, CAR is responsible for the attachment, internalization, and retrograde transport of canine adenovirus type 2 (CAV-2) vectors, which have found a niche in the mapping of neuronal circuits and gene transfer to treat and model neurodegenerative diseases. In this study, we used immunohistochemistry and immunofluorescence to document the global location of CAR in the healthy, young adult mouse brain. Globally, we found that CAR is expressed by maturing and mature neurons in the brain parenchyma and located on the soma and on projections. While CAR occasionally colocalizes with glial fibrillary acidic protein, this overlap was restricted to areas that are associated with adult neurogenesis.

## Introduction

Cell adhesion molecules (CAMs) are multifunctional proteins that, as the name suggests, also mediate interactions between cells or between cells and the extracellular matrix (ECM) (reviewed by Cavallaro and Dejana, 2011). While CAMs impact tissue structure, function, and cellular movement, they are also involved in cytoskeletal organization, contact inhibition, apoptosis, signaling, and transcriptional responses (reviewed by Gibson, 2001; Cavallaro and Dejana, 2011). Among the family of CAMs include a subset dubbed immunoglobulin superfamily (IgCAMs). In the central nervous system, some IgCAMs are indispensable for development and maintenance and several studies link their dysfunction with pathological conditions (reviewed Sakurai, 2017; Sytnyk et al., 2017).

The coxsackievirus and adenovirus receptor (CAR) belongs to the cortical thymocyte marker in *xenopus* CTX subfamily of IgCAMs (Chrétien et al., 1998; reviewed by Loustalot et al., 2016). As the name suggests, CAR was initially characterized for its role as an attachment molecule needed for binding and internalization of some coxsackievirus and adenoviruses (Bergelson et al., 1997, 1998; Carson et al., 1997; Tomko et al., 1997; Bergelson, 1999; Soudais et al., 2001). In contrast to most human adenovirus types, canine type 2 (CAdV-2 or CAV-2) appears to be dependent on CAR expression to infect cells and in particular neurons (Zussy et al., 2016; del Rio et al., 2019). CAR has the classical structure of IgCAMs: the extracellular domain (ECD) is composed of two Ig-like domains (D1 and D2) followed by a single-pass transmembrane domain (TM) and an intracellular domain (ICD) (Loustalot et al., 2016). In epithelial-like cells, the ECD and ICD of CAR interact with numerous intracellular and extracellular proteins (*ibid*). CAR is widely expressed in tight junction in the epithelial tissue in the adult mouse gastrointestinal tract, respiratory tract, kidney, and male reproductive system; it is also present in the liver, lymphatic system, skeletal muscle, and myocardial cells (Cohen et al., 2001; Shaw et al., 2004; Raschperger et al., 2006).

Like many prototypic CAMs, CAR engagement also induces signaling (Cavallaro and Dejana, 2011; Ortiz-Zapater et al., 2017). CAR overexpression leads to an increase in the phosphorylation/activation of GSK3β and Akt (Caruso et al., 2010). Moreover, CAR signaling influences E-cadherin levels and can increase MAPK activity (Farmer et al., 2009; Morton et al., 2013; Salinas et al., 2014). CAR engagement by viral proteins also activates the p44/p42 MAPK, JNK, and NF-κB pathways (Tamanini et al., 2006), suggesting that CAR is involved in pathways important for cellular homeostasis at the transcriptional level. The shedding of CAR’s ECD, likely with cell-type variations, is mediated either by α-secretase and metalloprotease 10 (Houri et al., 2013), or by β-site amyloid precursor protein-cleaving enzyme (BACE1) (Zhou et al., 2012). Then, a γ-secretase complex releases the ICD that translocates to the nucleus (Houri et al., 2013).

Several studies have also documented the presence of CAR in the rodent brain. As CAR expression is temporally regulated, very high levels of protein and mRNA were readily detected during embryonic brain development (Honda et al., 2000; Hotta et al., 2003; Venkatraman et al., 2005; Chen et al., 2019). CAR is observed from early developmental stages all along the neural tube, and as the secondary brain vesicles emerge, CAR immunoreactivity was mainly observed in cells and fibers in the telencephalon and diencephalon (Hotta et al., 2003; Chen et al., 2019). While CAR levels decrease significantly after birth, it is still readily detected in the adult mammalian brain, particularly in the blood–brain barrier, ependymal cells, and new born neurons in the hippocampus and olfactory bulb (Honda et al., 2000; Hotta et al., 2003; Venkatraman et al., 2005; Raschperger et al., 2006; Zussy et al., 2016; Salinas et al., 2017). Moreover, the CAR ECD interacts with several molecules involved in neuronal homeostasis including Agrin, a proteoglycan involved in synaptogenesis in the adult brain, and heparin-binding domain 2, a fibronectin that promotes neurite extension (Patzke et al., 2010). The ICD interacts with zonula-occludens 1, podocin, and PSD-95 (Excoffon et al., 2004; Yan et al., 2015). Accumulating evidence suggests that CAR is important for the trafficking of some of these proteins in non-neuronal cells (Farmer et al., 2009; Morton et al., 2013; Salinas et al., 2014) and with proteins involved in vesicle exocytosis at presynaptic termini in neurons (Wrackmeyer et al., 2019).

Of note, the genetic ablation of CAR expression in the mouse brain affects adult neurogenesis, synaptic content and function, and behavior (Zussy et al., 2016). Moreover, CAR loss of function had a greater impact on spatial memory and long-term plasticity in female mice. In addition, when healthy mice were injected in the peritoneal cavity with lipopolysaccharides (LPS) from gram-negative bacterial membrane, CAR levels decreased notably in the dentate gyrus (DG) (*ibid*). Chronic CAR loss was also seen in the DG of a mouse model of Alzheimer’s disease. These results link inflammation-induced posttranslational CAR loss in the hippocampus with changes in hippocampal plasticity and impaired cognition.

While numerous studies have focused on the role of CAR in the heart and epithelial tissues, there are only a handful of studies exploring its function in the brain. Detailing CAR’s regional and subcellular location is a primordial step to lay a foundation to understand its function in the healthy brain. In addition to its physiological role, CAR is responsible for the binding, internalization, and trafficking of canine adenovirus type 2 (CAV-2) (Soudais et al., 2001; Salinas et al., 2009). Therefore, understanding in which cells and in which regions CAR is expressed will allow more targeted approaches of using CAV-2 vectors to study and manipulate neuronal networks, and its potential for gene therapy and modeling neurodegenerative diseases (Soudais et al., 2004; Cubizolle et al., 2014; Beier et al., 2015; Schwarz et al., 2015; Hirschberg et al., 2017; Mestre-Francés et al., 2018).

## Materials and Methods

Five C57BL/6J and five C57BL/6N male mice between 6 and 8 weeks old were used in this study. Animal handling was conducted in accordance with the European Council directive (2010/63/EU) as well as in agreement with the Society for Neuroscience Policy on the Use of Animals in Neuroscience Research. The experimental design was approved by the Ethical Committee for Animal Testing Comité régional Languedoc-Roussillon.

Because genetic and phenotypic differences exist between C57BL/6J and C57BL/6N mice (reviewed by Morris et al., 2010), we used both strains to preclude a report biased on one strain. Animals were anesthetized with an overdose of ketamine/xylazine and then perfused transcardially with a saline solution (0.9% NaCl) followed by 50 ml of 4% paraformaldehyde prepared in phosphate buffer (PB), pH 7.4. The brains were removed and stored for 24 h in 4% paraformaldehyde. Afterward, the samples were washed in PB and cryopreserved in a solution containing 30% sucrose in PB. Finally, once the samples sank in the 30% sucrose solution they were frozen in Tissue Freezing Medium optimum cutting temperature (O.C.T.) (MicromMicrotech, TFM-5) and stored at −80°C. OCT blocks were cut in serial sagittal (1 brain each strain), horizontal (1 brain each strain), or coronal sections (3 brains each strain) (35 μm thick) using a cryostat and collected in a solution containing glycerol and ethylene glycol in PBS.

### Immunohistochemistry

Free-floating sections were rinsed with Tris–buffered saline (TBS) pH 7.4 and then incubated in a blocking solution containing 1% of gelatin from cold fish water skin (Sigma G7041), 1% bovine serum albumin (BSA) and 0.05% Triton X-100 in TBS for 1 h; after that, sections were incubated overnight at 4°C with the appropriate primary antibody/antibodies diluted in the blocking solution. The following primary antibodies were used for immunofluorescence: (1) a goat anti-CXADR (CAR) (1:100, R&D systems, AF2654, RRID:AB_2245567, Lot VFT0119071); (2) a rabbit anti-glial fibrillary acidic protein (GFAP) (1:1000, DAKO, Z0334, RRID:AB_10013382); (3) a mouse anti-SOX2 (1:200, ABCAM, ab171380, RRID:AB_2732072); (4) a mouse anti-NeuN (1:500, ABCAM, ab104224, RRID:AB_10711040); (5) a rabbit anti-doublecortin (DCX) (1:500, ABCAM, ab18723, RRID:AB_732011); and (6) a chicken anti-nestin (1:1000, ABCAM, ab134017, RRID:AB_2753197).

Following the incubation with primary antibodies, sections were rinsed with TBS and incubated with the appropriated biotinylated or fluorescent secondary antibody/antibodies diluted in the blocking solution for 1 h. The following secondary antibodies were used in this study: biotinylated horse anti-goat antibody: (1:500, Vector, BA9500, RRID:AB_2336123); Alexa Fluor^®^ 488 donkey anti-goat IgG (1:200, Molecular Probes A 11055, RRID:AB_2534102); Alexa Fluor^®^ 555 donkey anti-rabbit IgG (1:200, Molecular Probes A 31572, RRID:AB_162543); Alexa Fluor^®^ 647 donkey anti-mouse IgG (1:200, Molecular Probes A 31571, RRID:AB_162542); 4′,6-diamidino-2-phenylindole dihydrochloride (Sigma D8417); and Cy3 donkey anti-chicken IgG (1:200, Jackson ImmunoResearch, 703-165-155, RRID:AB_2340363.

The series incubated with biotinylated antibody were rinse in TBS and afterward with avidin–biotin complex (Vector Laboratories PK-6100, RRID:AB_2336819) for 1 h at room temperature. Once washed, the peroxidase reaction was visualized using 0.05% 3,3′-diaminobenzidine (Sigma, D5637) and 0.03% hydrogen peroxide.

Finally, sections were rinsed in TBS and mounted on SuperFrost Ultra Plus slides, dried at room temperature and counterstained using Harry’s hematoxylin, dehydrated and coverslipped with Eukitt (Sigma, 03989), and kept at room temperature or dried at room temperature and coverslipped with DAKO fluorescence mounting medium (DAKO, S3023) and kept at 4°C.

To test the specificity of the secondary antibodies, we omitted the primary antibodies in some sections while maintaining the rest of the procedures. All the control sections exhibited a lack of positive staining.

Immunofluorescence signals were visualized using a Zeiss LSM880 Airyscan laser-scanning microscope. The colorimetric signals were visualized using a Zeiss Axioimager Z2 microscope and a Retiga Q-imaging color camera (1920 × 1460 interlines, 4.64 μm pixel size). Images were adjusted for brightness and contrast by using ImageJ. Picture setup was achieved with Adobe Illustrator CS6. Full resolution was maintained until the micrographs were cropped and assembled, at which time they were adjusted to a resolution of 300 dpi.

The brain regions were identified using a mouse brain atlas (Franklin and Paxinos, 1997).

## Results

**FIGURE 1:**
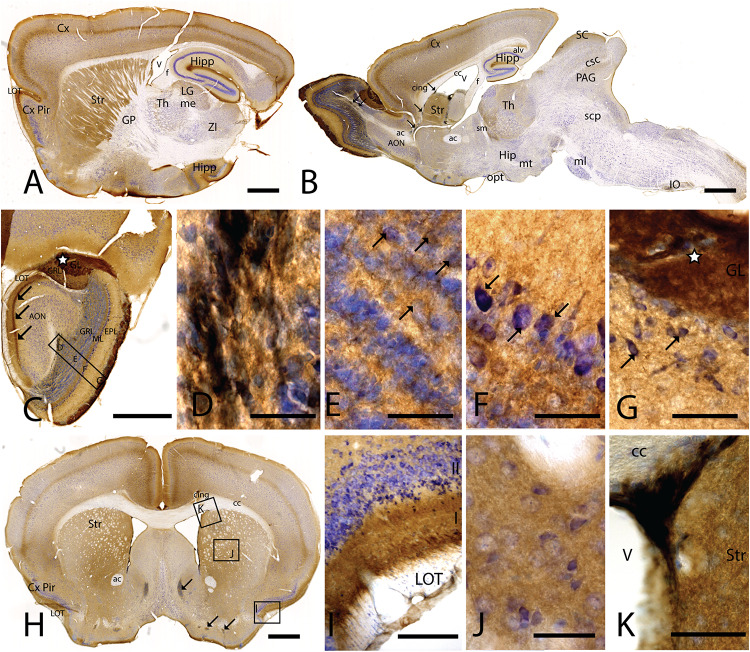
CAR expression in mouse brain. CAR immunoreactivity in brown counterstained with Harry’s hematoxylin in blue. **(A)** Sagittal lateral section of mouse brain. CAR is present in cortical areas—piriform cortex, isocortex, and hippocampus; and in non-laminated areas—striatum and thalamus. **(B)** Sagittal medial section of the mouse brain, where possible observe the presence of CAR mainly in the telencephalon and diencephalon, while the midbrain and rombencephalon showed CAR immunostaining only in specific regions. The cells of the RMS are indicated with arrows and also showed a high expression of CAR. **(C)** Coronal section of the olfactory bulb showing the presence of CAR in all the layers. It is noticeable the intense CAR immunolabeling present in layer I of the anterior olfactory nuclei are indicated with arrows. It is in the glomerulus of both the olfactory bulb and the accessory olfactory bulb where CAR staining is stronger (white star). **(D)** High magnification from **(C)** of the RMS. Due to the brown staining of CAR and the blue staining of the nuclei, in this photograph CAR expression is observed in black. CAR was located in the soma and in the neurites of the cells. **(E)** High magnification from **(C)** showing the granular cells layer. CAR was observed in some cellular somas (arrows) and also in the neuropil, which was detected as intense punctate. **(F)** High magnification of **(C)** showing the mitral cell layer. CAR is shown in the soma of some cells (arrows) and in the neuropil. **(G)** High magnification of **(C)** showing the external plexiform layer and the glomerulus of the olfactory bulb. The most streaking CAR expression was present in the glomerulus, where it was observed an intense extracellular labeling (white star), but it was also found in some cell soma of the periglomerular cells and in the neuropil of the external plexiform layer. **(H)** Coronal section of the telencephalon at the level of the striatum. Among other areas described before, CAR was observed along the cortical areas, the striatum, septum, Calleja islands, and subventricular zone. The arrows indicate the presence CAR immunolabeling in the Calleja islands. **(I)** High magnification of **(H)** showing a coronal section of the piriform cortex. The highest CAR expression is located in the layer I, while in the lateral olfactory bulb we can observe scattered CAR immunolabeled fibers, and layers II and III show staining in the neuropil. **(J)** High magnification of **(H)** showing the striatum. In this region, CAR was observed mainly in the neuropil. **(K)** High magnification of **(H)** where we can observe CAR expression in the dorso-lateral subventricular zone. The cells show an elongated shape and CAR was observed in the somas and neurites. Calibration bars: **(A,B)**: 1 mm, **(C)**: 100 c, **(D–G)**: 10 μm, **(H)**: 1 mm, **(I)**: 50 μm, **(J)**: 10 μm, **(K)**: 25 μm.

To study CAR expression in the mouse brain, we used a polyclonal goat antibody against the N-terminal which we previously demonstrated to be CAR specific (Zussy et al., 2016). In this study, we compared *C57BL/6J* and *C57BL/6N* mice. We did not observe differences in the CAR expression, and therefore, the description of CAR location applies to both strains. CAR expression was mainly, but not exclusively, seen in the telencephalon and diencephalon (Figures 1A,B). CAR immunoreactivity was readily detected in the cortex, olfactory bulb, striatum, septum, amygdala, hippocampus, some areas of the thalamus and hypothalamus, superior colliculus, and the brainstem.

### CAR Expression in the Telencephalon

In the telencephalon, the olfactory bulb and the accessory olfactory bulb contained the highest global level of CAR immunoreactivity. Using coronal, sagittal, and horizontal sections, we found CAR immunoreactivity in all the olfactory bulb layers, from the most inner layer to the outer layer (Figure 1C). CAR immunoreactivity was particularly intense in cells located in the rostral migratory stream (RMS) (Figures 1C,D): the cells had an elongated shape, and CAR was present in the soma and in the neurites. In the granular layer of the olfactory bulb, we observed CAR immunoreactivity in the neuropil and in some somas (Figure 1E arrows). A similar pattern was also observed in the mitral cells (Figure 1F) and plexiform layer (Figure 1G), where CAR was also present in the neuropil and in the soma of some mitral (arrows in Figure 1F) and periglomerular cells (arrows in Figure 1G).

The glomerulus in the olfactory bulb and the accessory olfactory bulb showed intense CAR immunoreactivity (white star in Figures 1C,G). CAR was also present in scattered fibers in the lateral olfactory tract (LOT) and with higher intensity in the layer I of the anterior olfactory nucleus (AON) (arrows in Figure 1C).

The pattern of CAR expression observed in the AON was also present in the olfactory tubercle and the rostral piriform cortex, where CAR immunoreactivity was present mainly in the layer I (Figures 1A,H,I). The pyramidal layer of the piriform cortex and olfactory tubercle did not show significant CAR immunoreactivity. CAR immunoreactivity was also observed in the islands of Calleja (arrows in Figure 1H). In the striatum (Figures 1A,B,H,J), high CAR immunoreactivity was present in the neuropil but low or absent in the fiber tracts (Figure 1J). By contrast, the globus pallidus showed faint CAR immunostaining in the neuropil (Figure 1A).

**FIGURE 2:**
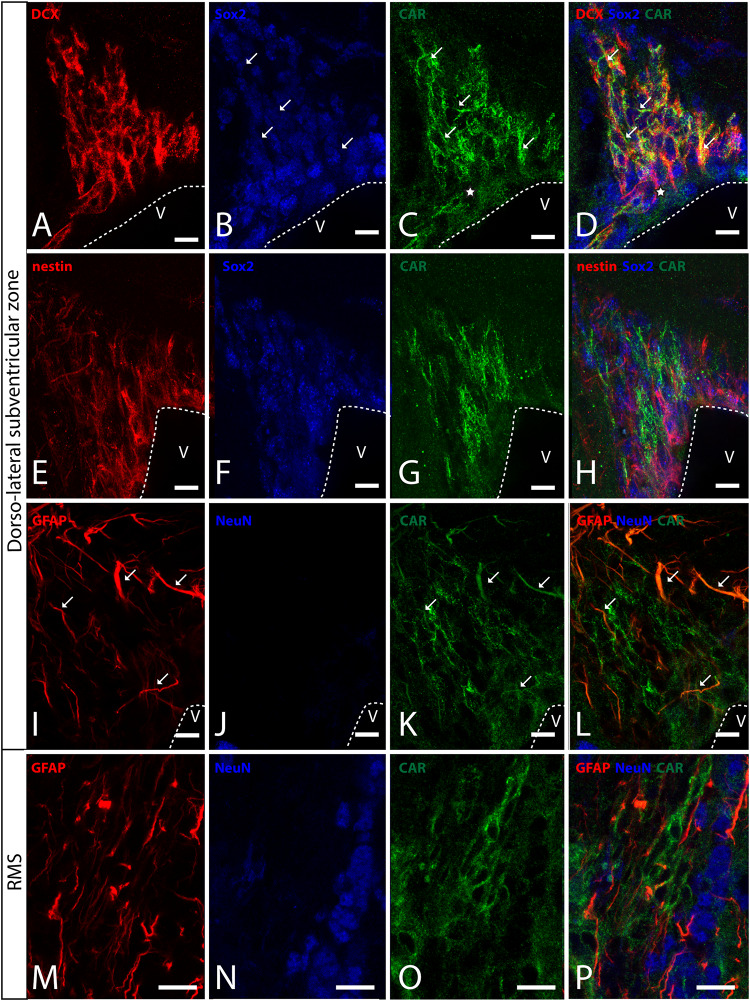
CAR expression in the sub-ventricular zone and the rostral migratory stream. **(A–D)** Colocalization of CAR (green), DCX (red), and Sox2 (blue) in the SVZ. Both CAR and DCX are present in the same cells (white arrows) that also express low levels of Sox2 (Z-stack 10 μm). **(E–H)** Colocalization of CAR (green) and nestin (red) in the SVZ. Nestin and CAR colocalization in some cells but not in all. And both CAR and nestin colocalize in those cells with lower levels of Sox2 (blue) (Z-stack 10 μm). **(I–L)** Colocalization of CAR (green) and GFAP (red) in the SVZ. Also, there was no presence of NeuN (blue) in the CAR^+^ cells (Z-stack 10 μm). **(M–P)** Expression of CAR (green), GFAP (red), and NeuN (blue) in the RMS. On the contrary that we observed in the SVZ, in the RMS there was a lack of CAR expression in the GFAP-ir fibers. As in the SVZ, CAR-ir cells were not NeuN immunoreactive (Z-stack 10 μm). Calibration bars: **(A–P)**: 10 μm.

In horizontal and sagittal sections, we observed CAR along the RMS (arrows in Figure 1B) and in the subventricular zone (SVZ), clearly shown in the coronal section (Figures 1H,K). To identity the CAR^+^ cells in the RMS and SVZ, we performed double and triple immunofluorescence labeling (Figure 2). In the SVZ, CAR (green Figure 2) colocalizes with markers for immature neurons DCX (Figures 1A–D) and SOX2 (Figures 2A–D). Of note, CAR was present mainly in the cells that showed low SOX2 immunoreactivity (arrows Figures 1B–D). This pattern was also the case for nestin positive cells in the SVZ (Figures 2E–H): higher CAR immunoreactivity in cells with lower levels of nestin immunoreactivity (Figures 2E,G,H). When we combined anti-CAR and anti-GFAP staining, we observed that the majority of GFAP-immunoreactive fibers (Figures 2I–L) in this area colocalized with CAR (white arrows in Figures 2I,K,L). However, not all the CAR-immunoreactive cells expressed GFAP (Figures 2I,K,L). Consistent with previous observations, CAR was not detected on the soma of NeuN-immunoreactive cells (mature neurons) (Figures 2M–P) in the areas surrounding the SVZ (Figures 2J,L).

This pattern of colocalization between CAR, DCX and nestin in the dorso-lateral SVZ was also observed in the ventro-medial SVZ and in the RMS (data not showed). However, in the RMS, GFAP (Figures 2M–P) and CAR (Figures 2M–P) were expressed in the same areas, but not in the same cells (Figure 2P). As described for the dorso-lateral SVZ, CAR did not colocalize with NeuN-Immunoreactive cells (Figure 2P).

**FIGURE 3:**
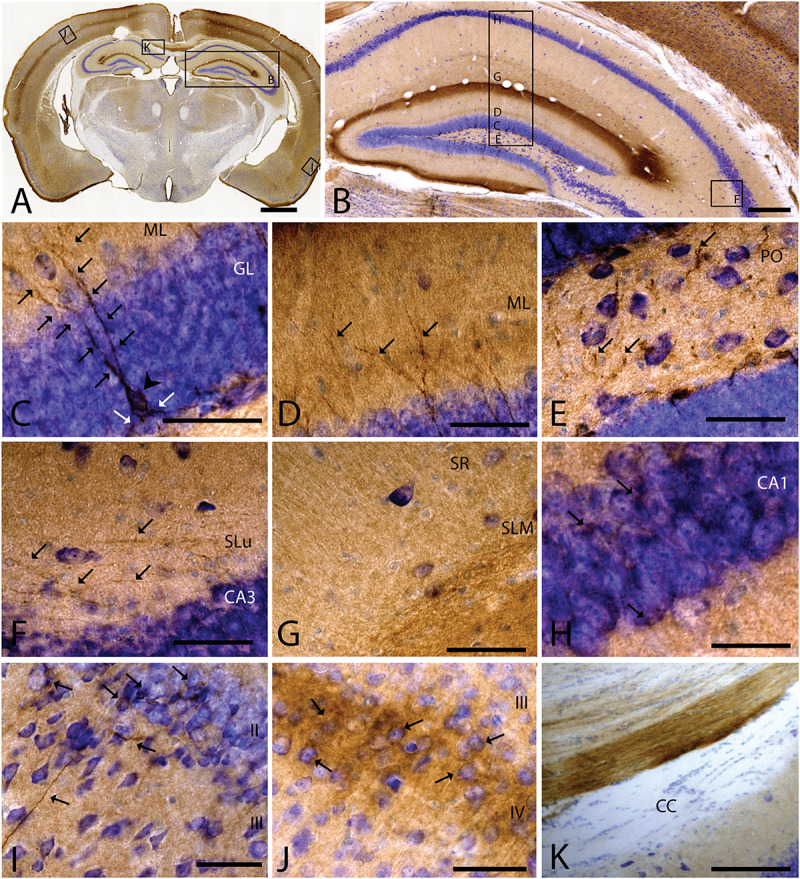
CAR expression in the hippocampus, cortical areas, and corpus callosum. CAR immunoreactivity in brown counterstained with Harry’s hematoxylin in blue. **(A)** Coronal section of mouse brain. CAR is present in cortical areas—piriform cortex, isocortex, and hippocampus; and in non-laminated areas—thalamus and hypothalamus. **(B)** High magnification of **(A)** showing the hippocampus. CAR is present in all layers of proper hippocampus and dentate gyrus. **(C)** Granular cell layer of the dentate gyrus high magnification of **(B)** showing the CAR-immunoreactive cells. The protein is present in the soma (arrow head) and in the apical and basal projections (arrows). **(D)** Molecular layer of the dentate gyrus, high magnification from **(B)**. In this layer, CAR is observed in the diffuse neuropil but also in the form of more intense dots in some the apical projection (arrows). **(E)** Presence of CAR^+^ fibers (arrows) in the hilus, high magnification from **(E)**. **(F)** CAR^+^ thin fibers in the stratum lucidus of CA3 (arrows), high magnification from **(B)**. **(G)** Detail of the stratum radiatum where CAR is present in the neuropil. **(H)** CAR expression in the CA1 pyramidal cell layer, high magnification from **(B)**. CAR is present in the cytoplasm of the cells (arrows). **(I)** CAR expression in the piriform cortex, which is present in thin fibers in layer III and cellular somas in layer II (arrows). **(J)** Layer IV of the isocortex showing the presence of CAR in the neuropil and in some cellular somas (arrows). **(K)** CAR-immunoreactive fibers in the medial portion of the corpus callosum. Calibration bars: **(A)**: 1 mm, **(B)**: 50 μm, **(D–K)**: 10 μm.

As we analyzed more caudal regions, we observed CAR immunoreactivity in cortical areas (Figures 3A,B). In the hippocampus, CAR was present in all layers of the DG and the proper hippocampus (Figure 3B). In the subgranular zone (SGZ) of the DG, we found the biggest population of CAR-immunoreactive cell bodies (Figures 3B,C): CAR-immunoreactive cells showed intense immunoreactivity in the soma (arrowhead in Figure 3C) and in the apical (black arrows in Figure 3C) and basal projections (white arrows in Figure 3C). In the molecular layer of the DG, CAR was present in the neuropil and in several branched fibers, many of them clearly belonging to the CAR-immunoreactive cells present in the SGZ (arrows in Figure 3D). CAR expression was also observed in the hilus (Figure 3E), in thin fibers running across the plexiform layer (arrows in Figure 3D). It was also possible to observe CAR-immunoreactive thin fibers in the stratum lacunosum-moleculare of the CA3 region (arrows in Figure 3F). In addition, the stratum lacunosum-moleculare of CA1 showed intense CAR immunostaining in the neuropil, being more intense in the area near to the molecular layer of the DG (Figures 3B,G). Weaker CAR immunoreactivity was also observed in the neuropil of the stratum radiatum of the CA1 (Figure 3G) and in the soma of the pyramidal cells of the CA1 (Figure 3H).

As mentioned above, other cortical areas also showed CAR immunoreactivity. In the caudal region of the piriform cortex, CAR immunoreactivity was present in layer I, in the soma of scattered cells in layer II, and in fibers along the layer III (arrows in Figure 3I). In the isocortex, CAR was observed in the neuropil along all the cortical layers, being more intense in layer I (Figure 3A) and layer IV; in the latter, CAR was present in the neuropil and in the membrane of some cell bodies (arrows in Figure 3J).

**FIGURE 4:**
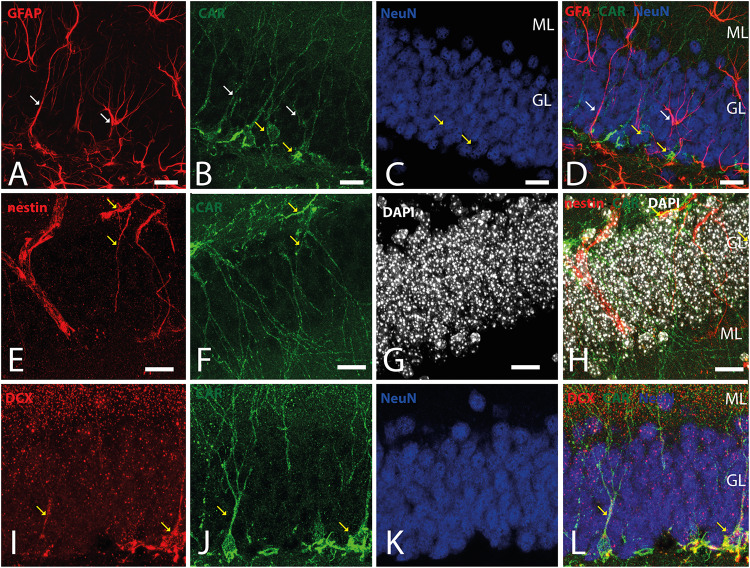
CAR expression in the subgranular layer of the dentate gyrus. **(A–D)** Expression of CAR (green), GFAP (red), and NeuN (blue) in the SGZ of the dentate gyrus. There are some fibers GFAP^+^ that also are CAR^+^ (white arrows). Some CAR^+^ cells are also expressing Neun (yellow arrows). **(E–H)** Colocalization of CAR (green), nestin (red), and DAPI (white) in the SGZ. Nestin and CAR colocalization in some cells (yellow arrows). **(I–L)** Colocalization of CAR (green), DCX (red), and DAPI (white) in the SGZ. Both CAR and DCX are present in the same cells (white arrows). Calibration bars: **(A–L)**: 10 μm.

Because CAR immunoreactivity was intense in the cells located in the SGZ, which is one place where adult neurogenesis occurs in the mouse brain, we performed double and triple immunolabeling to better identify these cells. By combining GFAP, NeuN, and CAR, we found that the majority of the CAR-immunoreactive soma do not colocalize with GFAP or NeuN (Figures 4A–D). However, there were a few fibers that were GFAP and CAR immunoreactive (white arrows in Figures 4A,B,D) and some cells expressing NeuN and CAR (yellow arrows in Figures 4B–D). Moreover, we found a handful of immunoreactive nestin and CAR fibers (yellow arrows Figures 4E–H). Similarly, co-immunolabeling with CAR and DCX showed overlapping immunoreactivity (yellow arrows in Figures 4I–L).

**FIGURE 5:**
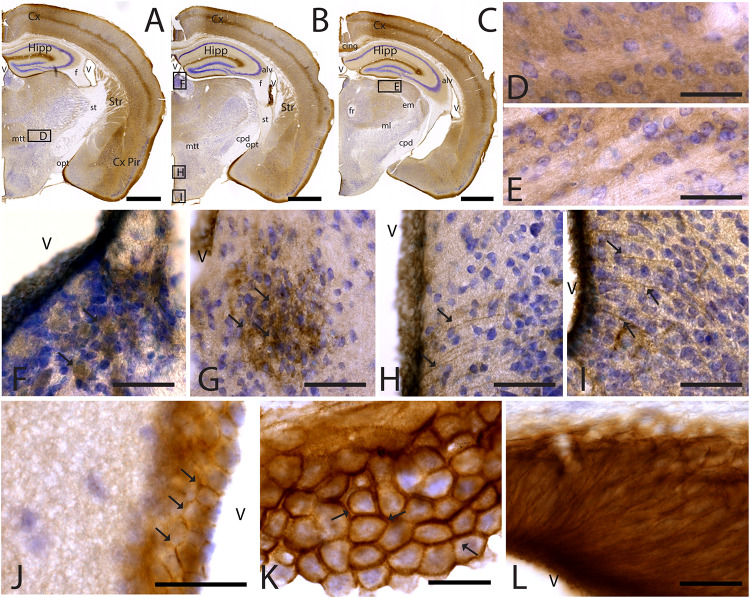
CAR expression in the thalamus and hypothalamus. CAR immunoreactivity in brown counterstained with Harry’s hematoxylin in blue. **(A–C)** Coronal section of mouse brain. CAR is present in the piriform cortex, isocortex, and hippocampus and in different nuclei of the thalamus and hypothalamus. **(D)** High magnification from **(A)**, ventral thalamus where CAR is present in the neuropil. **(E)** High magnification from **(C)** showing CAR immunoreactivity in the neuropil of the lateral geniculate nucleus. **(F)** CAR expression in the habenula, high magnification from **(B)**. **(G)** Detail of the preoptic area showing CAR expression (arrows). **(H)** Dorsal region of the medio basal hypothalamus showing the presence of CAR^+^ cells lining the 3rd ventricle and fibers in the adjacent regions (arrows). **(I)** Ventral region of the mediobasal hypothalamus showing the presence of CAR^+^ cells lining the 3rd ventricle and fibers in the adjacent regions. **(J)** CAR^+^ cells lining the 3rd ventricle in the thalamus. CAR is present in the cellular membrane (arrows). **(K)** Presence of CAR in the choroid plexus; arrows are showing the presence of protein in the cellular membrane. **(L)** CAR immunoreactivity in the cells of the subcommissural organ. Calibration bars: **(A–C)**: 1 mm, **(D–L)**: 10 μm.

In general, the big fiber tracts in the mouse telencephalon were CAR negative. However, we found exceptions along the rostro-caudal axis (Figures 1A,B, 3A,B, 5A–C) as we observed CAR-immunoreactive fibers in the cingulum bundle, the corpus callosum (Figure 3K), external capsule (Figures 1A,B), and the amigdalar capsule (Figures 5A–C). We also observed scattered CAR-immunoreactive fibers in some regions of the anterior commissure, alveus and stria terminalis, but not in the stria medullaris, fornix, or internal capsule (Figures 1A,B, 5A–C).

### CAR Expression in the Diencephalon

**FIGURE 6:**
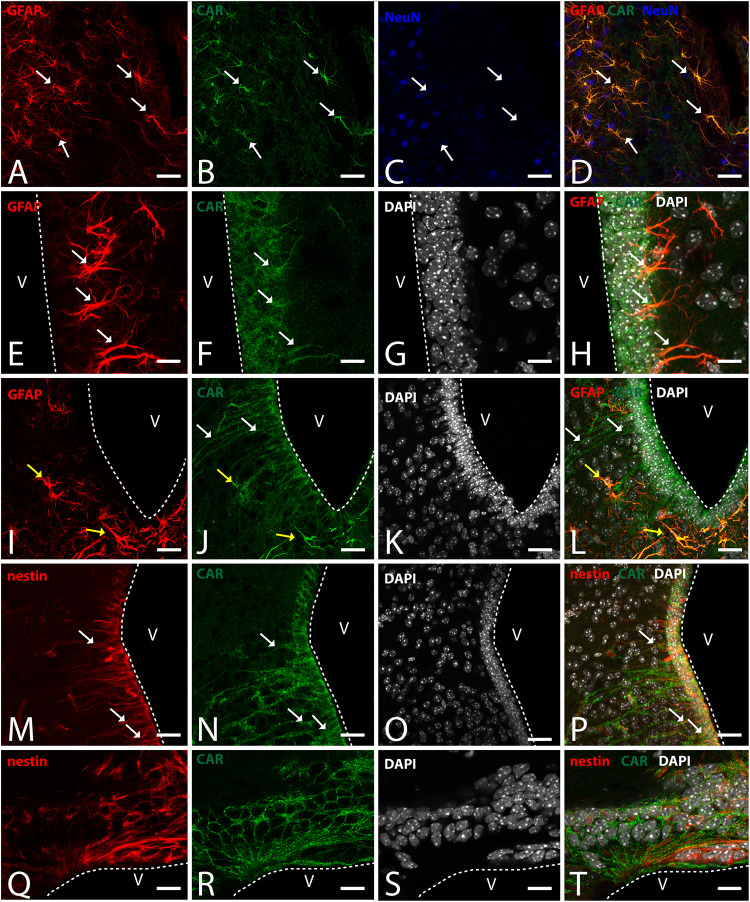
CAR expression in the hypothalamus. **(A–D)** Expression of CAR (green), GFAP (red), and NeuN (blue) in the medial preoptic area. White arrows indicating the colocalization of CAR and GFAP. **(E–H)** Expression of CAR (green), GFAP (red), and DAPI (white) in the mediobasal hypothalamus. Cells expressing CAR and GFAP indicated with white arrows. **(I–L)** Expression of CAR (green), GFAP (red), and DAPI (white) in the arquate nucleus. CAR-immunoreactive cells lining the ventricle and CAR cells also expressing GFAP indicated with yellow arrows in the parenchyma. **(M–P)** Expression of CAR (green), nestin (red), and DAPI (white) in the mediobasal hypothalamus. White arrows indicating the colocalization of CAR and nestin. **(Q–T)** Expression of CAR (green), nestin (red), and DAPI (white) in the subcommissural organ. Calibration bars: **(A–T)**: 20 μm.

As stated above, CAR immunoreactivity was also present in the thalamus and hypothalamus (Figures 5, 6). The fiber tracts in the diencephalon showed a general lack of CAR immunostaining in the optical tract, external medullary lamina of the thalamus, medial lemniscus, mammillary tract or fasciculus retroflexus (Figures 1A,B, 5A–C). However, we observed CAR-immunoreactive fibers in the diencephalon in the habenular commissure (Figures 1A,B).

In the thalamus, immunoreactivity was present in the neuropil of several nuclei including the nuclei of the anterior and lateral dorsal thalamus (Figures 5A–F), the lateral geniculate (Figure 5E) and reticular nuclei, the midline nuclei, and in some of the ventral nuclei (Figure 5D). In the habenula, CAR immunostaining appeared in what seemed to be fiber tracts running along the dorso-caudal axis (Figure 5F).

In the hypothalamus, CAR immunoreactivity was mainly present in the preoptic area and the rostral arcuate hypothalamic nucleus (arrows in Figure 5G), and along the ventricular zone of the mediobasal hypothalamus where immunoreactivity was strong in the ependymal cells lining the third ventricle and in fibers running from the ventricular wall toward the hypothalamic parenchyma (arrows in Figures 5H,I).

CAR immunoreactivity was also in the ependymal cells along the 3rd ventricle in the thalamus (Figures 5A–C,J), the lateral ventricles (Figures 1A,B,H), cerebral aqueduct, and 4th ventricle (data not shown). In the ependymal cells, CAR was present mainly in the plasma membrane (arrows in Figure 5K). CAR expression was also high in the choroid plexus along the rostro caudal axis (Figure 5K) and in the subcommissural organ (Figure 5L) where immunoreactivity was in the cytoplasmic membrane of ependymal cells (arrows in Figure 5K).

To identify the CAR-immunoreactive cells located in the mediobasal hypothalamus, we again perform double and triple immunolabeling (Figure 6). In the medial preoptic area, we found that CAR expression colocalizes with GFAP, but not with NeuN (Figures 6A–D). The white arrows in Figures 6A–D show CAR and GFAP-immunoreactive cells in this area. In the ventral area of mediobasal hypothalamus, the soma of the majority of the CAR-immunoreactive cells (Figure 6) lining the third ventricle did not colocalize with GFAP (Figures 6E–H), whereas some somas situated in the subventricular zone showed CAR-immunoreactive fibers running toward the hypothalamic parenchyma and those were also GFAP immunoreactive (white arrows in Figures 6E–H). In the arcuate nucleus, CAR-immunoreactive cells and fibers lining the ventricle were GFAP negative (white arrows in Figures 6I–L). However, in the same area in the parenchyma, a population of GFAP-immunoreactive cells were also CAR immunoreactive (yellow arrows in Figures 6I–L).

As expected in the dorsal area of mediobasal hypothalamus, we observed nestin-immunoreactive cells (Figures 6M–P) lining the 3rd ventricle as well as in fibers running toward the parenchyma. Although the CAR immunoreactivity had a similar distribution, these two proteins were mainly colocalized in the cells lining the ventricle and in some of the fibers in the parenchyma (white arrows in Figures 6M–P).

In the subcommissural organ, CAR and nestin immunoreactivity overlapped in some, but not all, cells (Figures 6Q–T).

### CAR Expression in the Caudal Regions of the CNS

**FIGURE 7:**
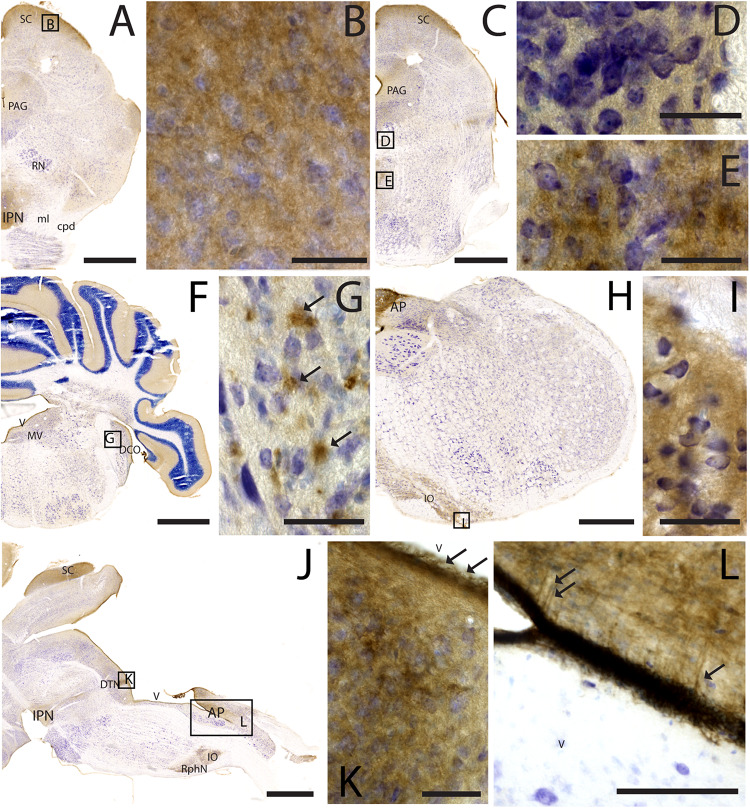
CAR expression in the midbrain and brainstem. CAR immunoreactivity in brown counterstained with Harry’s hematoxylin in blue. **(A)** Coronal section of mouse brain; CAR is present in the neuropil of the superior colliculus, the periaqueductal gray, and in the interpeduncular nucleus. **(B)** High magnification of **(A)** showing CAR immunostaining in the neuropil of the zonal layer in the superior colliculus. **(C)** Coronal section of mouse brain; CAR is present in the neuropil of the superior colliculus, the periaqueductal gray, and in the midline nuclei of raphe and reticular nuclei. **(D)** High magnification of **(C)** showing the presence of CAR in the neuropil of the midline raphe nuclei. **(E)** High magnification of **(C)** showing CAR immunoreactivity in the neuropil of the midline, corresponding to the tegmental reticular nucleus and raphe magnus. **(F)** Coronal section of mouse brain showing the presence of CAR immunostaining in the neuropil of the molecular layer of the cerebellar cortex, in the medial vestibular nucleus and dorsal cochlear nucleus. **(G)** High magnification of **(F)** showing the pattern of expression of CAR in the dorsal cochlear nucleus. **(H)** Coronal section of mouse brain showing CAR expression in the caudal rhombencephalon, in the area postrema, and inferior olive complex. **(I)** High magnification of **(H)** showing the presence of CAR immunostaining in the neuropil of the inferior olive complex. **(J)** Sagittal section of a mouse brain showing the presence of CAR immunolabeling in the superior colliculus, interpeduncular nucleus, in the dorsal tegmental nucleus, area postrema, inferior olive complex and raphe nuclei. **(K)** High magnification of **(J)** showing the presence of CAR in the neuropil of the dorsal tegmental nucleus and the ependymal cell lining the 4th ventricle (arrows). **(L)** Magnification of **(J)** showing the 4th ventricle and the area postrema. CAR is present in the neuropil and in the projections toward the parenchyma. Calibration bars: **(A–B)**: 1 mm; **(C–E)**: 5 μm; **(F)**: 1 mm; **(G)**: 5 μm; **(H)**: 1.5 mm; **(I)**: 5 μm; **(J)**: 1 mm; **(K)**: 5 μm; **(L)**: 15 μm.

As noted above, CAR immunoreactivity was globally lower in the midbrain and rhombencephalon. In general, the fiber tracts in midbrain were CAR negative (Figure 7). We found CAR in the neuropil of the zonal layer of the superior colliculus (Figures 7A,B), all along the periaqueductal gray (Figure 7A), and in the ependymal cells of the cerebral aqueduct. The substantia nigra showed a faint neuropil staining, mainly in the pars compacta. Caudally at the tegmentum, CAR immunoreactivity was found in the neuropil of the nuclei situated in the midline (Figure 7C), corresponding with the raphe formation (Figures 7C,D) and the tegmental reticular nucleus (Figures 7C,E). The nuclei of the lateral lemniscus showed a pattern of CAR immunoreactivity similar to that of the lateral geniculate nuclei.

In the rhombencephalon, notable CAR immunoreactivity was observed in the dorsal cochlear nucleus (Figures 7F,G), with an expression pattern similar to that of the habenula (arrows in Figure 7G). The nuclei situated in the medio-dorsal area of the rhombencephalon along the rostro-caudal axis showed faint CAR immunostaining in the neuropil, while in the locus coeruleus, inferior olive complex (Figures 7H,I), and in the area postrema, the labeling in the neuropil was more intense (Figures 7J,L). There was also CAR immunoreactivity in the neuropil of the dorsal motor nucleus of the vagus nerve and the hypoglossal nucleus.

In sagittal sections, we found a population of CAR-immunoreactive cells located in the caudal region of the 4th ventricle, area postrema, with the somas situated in the ventricular zone and the projections toward the parenchyma (Figures 7J,L). This pattern resembled that of in the hypothalamus (data not shown). The cerebellum showed a faint CAR immunostaining in the neuropil of the molecular layer (Figure 7F).

## Discussion

IgCAMs play a role in several processes during brain development and in the mature brain, which include dendritic spine development, neurite outgrowth, axon guidance and fasciculation, adult neurogenesis and synapse remodeling (reviewed by Gibson, 2001). This study characterized the expression of CAR throughout the adult mouse brain and will contribute to understanding its physiological role. Of note, the anti-CAR antibody used here (R&D systems, AF2654, RRID:AB_2245567, Lot VFT0119071) did not show immunoreactivity in brain sections from CAR-CNS knockout mice (see Supplementary Figure 1) (Zussy et al., 2016). We used C57BL/6J and C57BL/6N mice because previous studies have shown different pattern expression of several genes and proteins (Morris et al., 2010), metabolism and inflammatory response (Simon et al., 2013) and emotional responses to social stress (Hovatta et al., 2005; Bryant et al., 2008; Matsuo et al., 2010; Kulesskaya et al., 2011; Chen et al., 2019). In these mice, we found no striking differences in the intensity or pattern of CAR immunoreactivity.

While CAR levels decrease notably after birth, CAR is readily detected in cells, fibers, and in the neuropil of several brain areas in the adult brain. We observed CAR immunoreactivity in both cortical areas and nuclei all along the brain, with the olfactory bulb and the accessory olfactory bulb showing the highest immunoreactivity. Globally, we concluded that CAR is expressed primarily by maturing and mature neurons in the brain parenchyma. Of note, while CAR occasionally colocalizes with GFAP, it remained limited to the boundary of the 3rd ventricle, the SVZ (areas that are associated with adult neurogenesis) and specific populations located in the hypothalamus, an area that may generate new neurons in the adult mouse brain and is associated with hyperplasticity (Parkash and Kaur, 2007; Bolborea, 2011).

In the RMS, SVZ, and SGZ, we found CAR expression in accordance with previous studies (Hotta et al., 2003; Venkatraman et al., 2005; Zussy et al., 2016; Salinas et al., 2017; Chen et al., 2019; Wrackmeyer et al., 2019). The partial colocalization of CAR with GFAP, nestin and SOX2 in the SVZ demonstrates that CAR is express during adult neurogenesis, as was propose previously in the SGZ of the DG (Zussy et al., 2016).

In the SVZ, we found CAR in two different cell populations: in SOX2^+^, GFAP^+^ and nestin^+^ cells, which is consistent with expression of CAR in stem cells and type B1 cells (Codega et al., 2014; Fuentealba et al., 2015; Llorens-Bobadilla et al., 2015), and DCX^+^ cells, which correspond to type A or neuroblasts (reviewed by Ming and Song, 2011). The main difference between those two CAR-immunoreactive populations is the fact that the cells expressing SOX2, nestin, or GFAP in the SVZ only show a partial colocalization with CAR, while the DCX^+^ cells showing a clear colocalization and also higher levels of CAR expression. These differences are clearer in the RMS where CAR^+^/DCX^+^ cells were readily detected, while CAR-immunoreactivity is absent in the GFAP^+^ cells. In the RMS, the migrating neuroblasts are surrounded by glial cells that serve as a scaffold for the neuroblast migration (Lois and Alvarez-Buylla, 1994; Snapyan et al., 2009; Kaneko et al., 2010). The differences between the SVZ and the RMS could be due to two GFAP^+^ cell populations, but this needs a more detailed study. In the SGZ, CAR also colocalizes with DCX, but in contrast to the SVZ, CAR was essentially absent in the GFAP^+^ cells. These results agree with previous results showing the colocalization of CAR and PSA-CAM in this area (Salinas et al., 2017), suggesting that CAR plays a role at different stages of neuronal maturation and in different neurogenic areas. It is tempting to speculate that in the SGZ, CAR regulates network development and integration, whereas in the SVZ, it is involved in the migration of NPCs along the RMS and possibly also during differentiation and integration.

We show that CAR is present in an eclectic combination of regions in the adult mouse brain. In general, CAR is expressed in the neuropil of numerous adult brain regions. The cortex is present in all the layers but is more prominent in layers I and IV. PSA-NCAM is also in the cortical neuropil (Varea et al., 2005), which is involved in brain plasticity. Furthermore, in the thalamus and brainstem, CAR expression is similar to that of PSA-NCAM, where it is in the neuropil of several nuclei and characterized by structural plasticity and related to memory and learning (Mazzetti et al., 2007; Quartu et al., 2010).

Coxsackievirus and adenovirus receptor immunoreactivity in the preoptic area and hypothalamic median eminence-arcuate region had not been previously reported. The hypothalamic median eminence-arcuate region may be an additional site adult neurogenesis as a handful of studies suggested that α2 tanycytes may function as stem-like cells and that β tanycytes are lineage-restricted cells with limited proliferative potential (reviewed by Yoo and Blackshaw, 2018). In this area, CAR colocalizes with GFAP and nestin indicating that it is present in α1and α2 tanycytes. CAR was also present in the arcuate region in the cells lining the ventricle that were GFAP negative, and therefore possible β tanycytes. There are also other CAR-immunoreactive populations located in the preoptic area and the rostral arcuate hypothalamic nucleus that colocalize with GFAP. Previous studies have shown colocalization of PSA-NCAM and GFAP in tanycytes and in cells present in the parenchyma of the preoptic area and arcuate nucleus. Here, PSA-NCAM expression changes are related with a high capacity for neuroplastic changes in the adult rodent brain and the modulation of gonadotropin release induced by neuroendocrine signals (Parkash and Kaur, 2007; Bolborea, 2011). CAR’s presence in these regions and in the GFAP^+^ populations opens the door to study the changes in CAR expression induced by different neuroendocrine signals. Our previous results showed that under peripheral inflammatory conditions (peritoneal LPS injection), there is a posttranslational CAR loss that correlates with changes in hippocampal plasticity and neurogenesis (Zussy et al., 2016), which makes the preoptic area and the rostral arcuate hypothalamic nucleus candidates for further studies of the impact of inflammation on CAR levels. Moreover, the GFAP-immunoreactive population in the hypothalamus has been studied in relation with reactive astrogliosis in the hypothalamus in response to diet in rodents; chronic low-grade inflammation in peripheral tissues due to a fat-rich diet induced hypothalamic inflammation, an increase of proinflammatory cytokines such as TNF and IL-1β and an increase in gliosis (reviewed by Seong et al., 2019). CAR levels in the hypothalamic median eminence-arcuate region could also be affected by a proinflammatory environment induced by disease or poor diet. It is also noteworthy that CXADR (the gene coding for CAR) contains an estrogen response element in its promoter (Lucas et al., 2003). Clearly, further studies are needed to characterize the role of CAR in this enigmatic region.

Consistent with previous studies, we found CAR in the epithelial cells of the adult choroid plexus and in the neuroepithelium surrounding the ventricles (Hotta et al., 2003; Chen et al., 2019), as well as in the aqueduct and in the subcommissural organ. In the epithelial cells, CAR was on the basolateral surface and low or absent on the apical surface, supporting the hypothesis that it may participate in the polarization of the neuronal stem cell niche favoring radial, asymmetric division of progenitors (reviewed by Hauwel et al., 2005). The choroid plexus and the subcommissural organ are secretory tissues responsible for producing the cerebrospinal fluid, as an interface between the blood and the CNS, and involved in the regulation of the adult neurogenesis. The choroid plexus is a port of entry for immune cells and thus a potential site for communication between the immune system and the CNS (reviewed by Lun et al., 2015). Recent studies have demonstrated that the choroid plexus expresses interferons which facilite the *trans-*epithelial passage of leukocytes (Peralta et al., 2017). CAR plays an active role in *trans-*epithelial passage in other epithelial monolayers, where it is phosphorylated in response to TNF (Morton et al., 2016). Therefore, in the choroid plexus CAR may play a similar role.

The study of connectivity and wiring in the brain is a challenge. Moreover, linking expression of proteins on projections and terminals to that of the soma needs specific tools. Traditionally, it was done using chemical tracer, molecules injected in the projection site and transported to the soma or in the other way around, helping us to identify the origin of the projections in specific areas. In recent years, viral vector has been used as tracers. CAV-2 vectors preferentially transduce neurons (Soudais et al., 2004; Cubizolle et al., 2014; Beier et al., 2015; Schwarz et al., 2015; Hirschberg et al., 2017; Mestre-Francés et al., 2018) and are widely used in brain studies. It is important to note that, to the best of our knowledge, CAV-2 depends on CAR to bind and be internalized in neurons. This CAR-tropic nature of CAV-2 is further supported by the lack of infection of cells in CAR CNS-KO mice (Zussy et al., 2016). Different *in vitro* assays have characterized the CAR-dependent mechanisms regulating CAV-2 entry and transport in primary rodent motor neurons (Salinas et al., 2009; Henaff et al., 2011; Simão et al., 2015), and how it occurs in pH neutral endosomes, which allows long-range transport in an environment that precludes conformational changes of the capsid and endosomal escape. Numerous studies have shown that CAV-2 vectors can target different subpopulations of neurons in the brain in rodents, dogs, and non-human primates (Soudais et al., 2004; Cubizolle et al., 2014; Beier et al., 2015; Schwarz et al., 2015; Hirschberg et al., 2017; Mestre-Francés et al., 2018). CAV-2 retrograde transport is also noteworthy: for example, from the striatum to the soma of dopaminergic neurons in the substantia nigra pars compacta, thalamic neurons, and cortical neurons (layer IV) of the ipsilateral and contralateral isocortex (Soudais et al., 2004; Hnasko et al., 2005; Kremer, 2005; Junyent and Kremer, 2015; del Rio et al., 2019). In addition to retrograde transport, infection of neurons at the site of injection is also robust. When a CAV-2 vector carrying GFP gene is injected in the striatum, one detects dense GFP signal in the soma of striatal neurons (Soudais et al., 2004; Cubizolle et al., 2014; Beier et al., 2015; Schwarz et al., 2015; Hirschberg et al., 2017; Mestre-Francés et al., 2018). Another example is the SOX2-positive cells lining the lateral ventricles that are infected by CAV-2 when it is injected into ventricles/cerebral spinal fluid (Salinas et al., 2017). Here is where the knowledge of CAR expression in the adult brain is an important factor to help in the study of the brain connectivity in healthy or pathological models. CAV-2 vectors are able to transduce different types of neurons, motor, sensory, parasympathetic, GABAergic, cholinergic, norepinephrine, and dopamine neurons (Hnasko et al., 2006; Salinas et al., 2009; Beier et al., 2015; Schwarz et al., 2015; Li et al., 2016; Uematsu et al., 2017), but the preference for neuronal subtypes is not fully characterized. The presence of CAR has been described in the presynaptic fraction of synaptosome preparations from adult mouse, prosimian, monkey, and human brains (Zussy et al., 2016; Mestre-Francés et al., 2018). *In vitro*, it is clear that CAV-2 can infect neurons by binding to CAR at axon terminals (Salinas et al., 2009, 2010); however, there is a lack of studies in relation with CAR distribution and density along the axons *in vivo* and its presence in the soma of different neuronal populations. The fact that CAV-2 vectors can efficiently enter a neuron via presynaptic termini does not, *a priori*, exclude other entry sites (Schwarz and Luo, 2015). However, anecdotal data suggest that if CAV-2 is taken up via axon *en passant*, it is not robust.

## Conclusion

In conclusion, we generated a global description of CAR immunoreactivity in the male mouse brain. Our study is the basis for comparative studies with other mammals and to further explore the function of CAR in the neurogenic niches of the SVZ, SGZ, and in relation with plasticity in different areas of the brain in the healthy, diseased, or proinflammatory-challenged brain.

## Data Availability Statement

All datasets generated for this study are included in the article/Supplementary Material.

## Ethics Statement

The animal study was reviewed and approved by the Comité Régional Languedoc-Roussillon.

## Author Contributions

ID-R and EK were responsible for the concept, design, supervision of the study, and contributed to the initial draft of the manuscript. AW and ID-R were responsible for the acquisition. ID-R was responsible of the analysis, and interpretation of histological data.

## Conflict of Interest

The authors declare that the research was conducted in the absence of any commercial or financial relationships that could be construed as a potential conflict of interest.

## Abbreviations

Ac: anterior commissure
Alv: alveus
AON: anterior olfactory nucleus
AP: area postrema
Cc: corpus callosum
Cing: cingulum bundle
Cpd: cerebellar peduncle
Csc: superior colliculus commissure
Cx: cortex
Cx: pir piriform cortex
DCO: dorsal cochlear nucleus
DTN: dorsal tegmental nucleus
Em: external medullary lamina of the thalamus
EPL: external plexiform layer
F: fimbria
GL: glomerular layer
GL: granular cells layer hippocampus
GP: globus pallidum
GRL: granular cells layer of olfactory bulb
H: hilus
Hip: hipothalamus
Hipp: hippocampus
IO: inferior olive complex
IPN: interpeduncular nucleus
LOT: lateral olfactory tract
Ml: medial lemniscus
ML: molecular layer
Mtt: mammillothalamic tract
MV: medial vestibular nucleus
Opt: optical tract
PAG: periaqueductal gray
Po: polymorphic layer
RN: red nucleus
RphN: raphe nuclei
SC: superior colliculus
Scp: superior cerebellar peduncles
SLM: stratum lacunosum-moleculare
SLU: stratum lucidum
Sm: stria medullaris
SML: stratum molecular
SR: stratum radiatum
Str: striatum
Th: thalamus
V: ventricle.
