# Supplementary material for: Location of the Cell Adhesion Molecule “Coxsackievirus and Adenovirus Receptor” in the Adult Mouse Brain
Source: Front Neuroanat. 2020 Jun 4;14:28. doi: 10.3389/fnana.2020.00028 (PMC7287018; doi:10.3389/fnana.2020.00028)
Supplement: Supplementary file 1 [file Data_Sheet_1.PDF]

Supplementary Material

1 Supplementary material and methods

We used one male mouse C57BL/6N WT and one C57BL/6N CAR-CNS KO (Zussy et al., 2016). The animals were anesthetized with an overdose of ketamine/xylazine and then perfused transcardially with a saline solution (0.9% NaCl) followed by 50 ml of 4% paraformaldehyde prepared in phosphate buffer (PB), pH 7.4. The brains were removed and stored for 24 hours in 4% paraformaldehyde. Afterwards the samples were washed in PB and cryopreserve in a solution containing 30% sucrose in PB. Finally, they were frozen in Tissue Freezing Medium® optimum cutting temperature (O.C.T.) (Microm Microtech, TFM-5) and stored at -80°C. OCT blocks were cut in serial coronal sections (35 µm thick) using a cryostat and collected in a solution containing glycerol and ethylene glycol in PBS.

1.1 Immunohistochemistry

Free-floating sections were rinsed with Tris buffer pH 7.4 (TBS) and then incubated in a blocking solution containing 1% of gelatin from cold fish water skin (Sigma G7041), 1% bovine serum albumin (BSA) and 0.05% Triton X-100 in TBS for 1 hour; after that, sections were incubated overnight at 4°C with a goat anti-CXADR (CAR) (1:100, R&D systems, AF2654, RRID:AB_2245567, Lot VFT0119071) diluted in the blocking solution.

The day after, sections were rinsing with TBS and incubated with biotinylated horse anti-goat antibody: (1:500, Vector, BA9500, RRID:AB_2336123) secondary antibody diluted in the blocking solution for 1 h. Afterwards the sections were wash in TBS and finally incubated with avidin-biotin-complex (Vector Laboratories PK-6100, RRID:AB_2336819) for 1 h at room temperature. Once washed, the peroxidase reaction was visualized using 0.05% 3,3′-diaminobenzidine (Sigma, D5637) and 0.03% hydrogen peroxide.

Sections were rinsed in TBS and mounted on SuperFrost Ultra Plus® slides, dried at room temperature and counterstained using Harry's hematoxylin, dehydrated and coverslipped with Eukitt.

The signals were visualized using a Zeiss Axioimager Z2 microscope and a Retiga Q-imaging color camera (1920 X 1460 interlines, 4.64 µm pixel size).

The images were adjusted for brightness and contrast by using ImageJ. Picture set up was achieved with Adobe Illustrator CS6. The full resolution was maintained until the micrographs were cropped and assembled, at which were adjusted to a resolution of 300 dpi.

The brain regions were identify using a mouse brain atlas (Franklin and Paxinos, 1997).

2 Supplementary Figures

When we incubated the anti-CAR antibody and secondary anti-goat Ab with brain slices from KO mice, we also included control wild type brain slices in the assay. We found the same CAR staining in the wild type tissue as that presented throughout the manuscript.

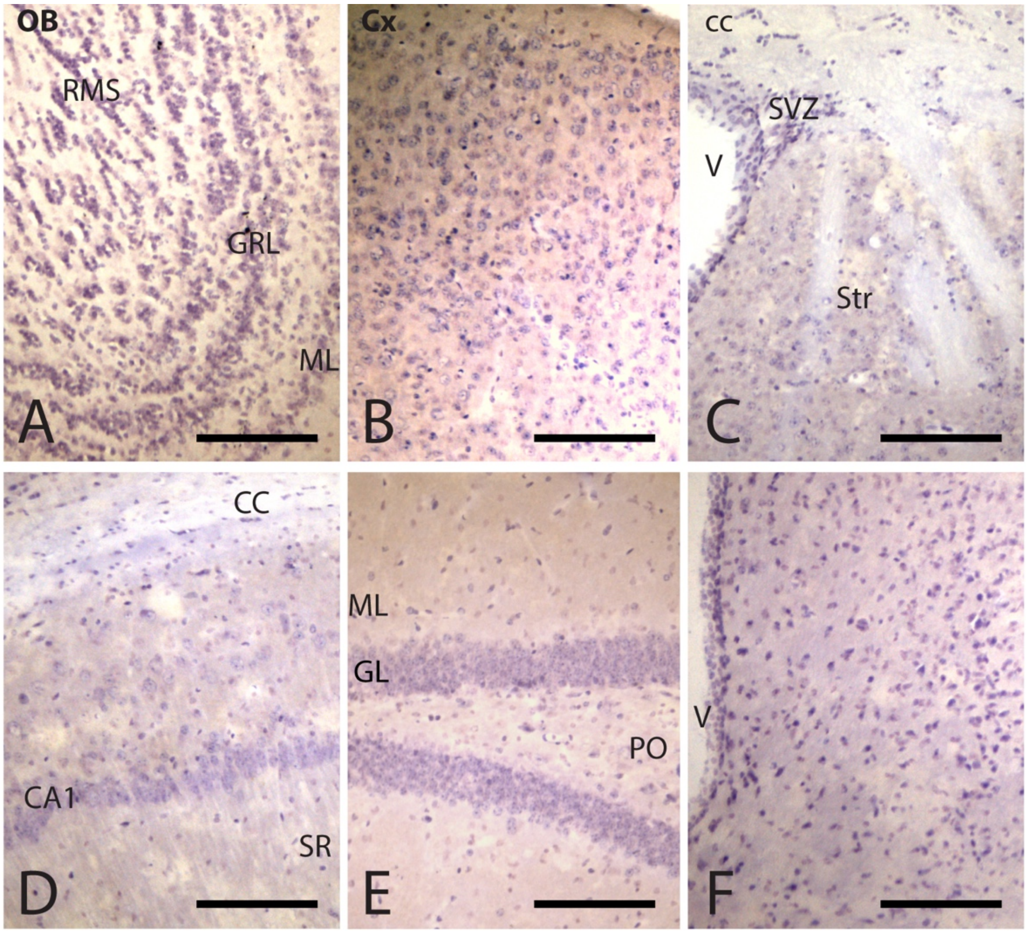

Supplementary Figure 1. CARCNS KO mice. IHC against CAR using AF2654, lot VFT0119071 (dilution 1:100). A) the olfactory bulb; B) the isocortex; C) the SVZ and dorsal striatum; D) the CA1 region of the hippocampus and the commissural fibers; E) the dentate gyrus of the hippocampus; F) the hypothalamus at the level of the arcuate nucleus. Calibration bars: 25 um
